# Proteomics Revealed Distinct Responses to Salinity between the Halophytes *Suaeda maritima* (L.) Dumort and *Salicornia brachiata* (Roxb)

**DOI:** 10.3390/plants9020227

**Published:** 2020-02-10

**Authors:** Jenifer Joseph Benjamin, Begoña Miras-Moreno, Fabrizio Araniti, Hajar Salehi, Letizia Bernardo, Ajay Parida, Luigi Lucini

**Affiliations:** 1 Department of Plant molecular Biology, MS Swaminathan Research Foundation, III Cross Street, Taramani Institutional Area, Taramani, Chennai 600113, India; jeniferjoseph@mssrf.res.in; 2 Council for Agricultural Research and Economics—Research Centre for Genomics and Bioinformatics (CREA-GB), via San Protaso 302, 29017 Fiorenzuola d’Arda, PC, Italy; 3 Department of AGRARIA, University “Mediterranea” of Reggio Calabria, I-89124 Reggio Calabria, Italy; fabrizio.araniti@unirc.it; 4 Laboratory of Plant Cell Biology, Department of Biology, Bu Ali Sina University, Hamedan 65178-38695, Iran; h.salehi92@basu.ac.ir; 5 Department for Sustainable Food Process, Università Cattolica del Sacro Cuore, 29122 Piacenza, Italy; l_bernardo@libero.it (L.B.); luigi.lucini@unicatt.it (L.L.); 6 Institute of Life Sciences, Department of Biotechnology, Government of India, Bhubaneswar 10, Odisha 751023, India

**Keywords:** salt stress, plant proteomics, Chenopodiaceae, photosynthesis, signaling, osmotic stress

## Abstract

Plant resistance to salinity stress is one of the main challenges of agriculture. The comprehension of the molecular and cellular mechanisms involved in plant tolerance to salinity can help to contrast crop losses due to high salt conditions in soil. In this study, *Salicornia brachiata* and *Suaeda maritima*, two plants with capacity to adapt to high salinity levels, were investigated at proteome level to highlight the key processes involved in their tolerance to NaCl. With this purpose, plants were treated with 200 mM NaCl as optimal concentration and 500 mM NaCl as a moderate stressing concentration for 14 days. Indeed, 200 mM NaCl did not result in an evident stress condition for both species, although photosynthesis was affected (with a general up accumulation of photosynthesis-related proteins in *S. brachiata* under salinity). Our findings indicate a coordinated response to salinity in both the halophytes considered, under NaCl conditions. In addition to photosynthesis, heat shock proteins and peroxidase, expansins, signaling processes, and modulation of transcription/translation were affected by salinity. Interestingly, our results suggested distinct mechanisms of tolerance to salinity between the two species considered, with *S. brachiata* likely having a more efficient mechanism of response to NaCl.

## 1. Introduction

Soil salinity is one of the primary causes of crop losses worldwide. Every year, about 1.5 million hectares of agriculture lands are affected by high salinity and rendered unsuitable for crop production [1,2]. Salinity affects about 20% of irrigated land primarily due to improper agricultural practices [3]. Many studies on current saltwater and freshwater interactions in coastal aquifers have demonstrated the increasing global problem of saltwater intrusion [4,5]. Soil salinization severely affects agricultural productivity, because most crops cannot tolerate sodium ion (Na^+^) concentrations greater than 150–200 mM NaCl [6,7]. Most of the plants can adapt to low or moderate saline conditions, but their growth is severely reduced above 200 mM NaCl. Salinity imposes an initial osmotic effect and a subsequent ionic effect, reducing the ability of plants to uptake water and micronutrients [8]. Understanding the molecular and cellular mechanisms of salt tolerance are critical to help improve plant growth and productivity under saline conditions.

Halophytes are efficient in adaptation and have a well-orchestrated mechanism in place to deal with salinity stress [9,10]. They are able to complete their life cycle in saline conditions. The ability to regulate the amount of salt translocated to the shoot through the transpiration stream might be a determining factor in salt tolerance [8]. Halophytes can attain this Na^+^ and Cl^−^ (chloride) exclusion under high saline conditions [11]. In mangroves, Na^+^ exclusion from the xylem is carried out through anatomical adaptations that reduce or prevent apoplastic movement of solution from outside the roots to the xylem; this ensures that cellular membranes and transporters can determine the ions which pass into the xylem [12]. In order to decrease the net uptake of salt ions to the shoots, halophytes display reduced stomatal opening which leads to the generation of ROS. The capability of halophytes to handle with ROS was studied in *Eutrema parvulum*, a close relative of *Arabidopsis thaliana* [13].

Numerous studies are being performed in diverse halophytes to understand and identify the genes responsible for salt tolerance [14,15]. Despite various salt stress-responsive genes have been reported, a complete understanding of salt tolerance mechanism still remains elusive. Halophytes capability to adapt to high salinity levels is often ascribed to regulation at both transcriptional and proteomic levels [16]. Proteins act as a major stimulator of plant stress response. Proteins not only act as enzymes but also as a key component in the transcription and translation processes, thereby plant stress response genes are regulated both at RNA and protein level [17]. However, stress response genes expressed at mRNA and protein level cannot be correlated due to the post-transcriptional and post-translational modifications in the former [18]. Several salt stress responsive proteins are expressed at different cellular functions such as signal transduction, regulation of carbohydrate, nitrogen and energy metabolism, RNA and protein synthesis, ROS regulation and redox homeostasis. Hence, quantitative proteomics is a prominent tool to be applied to study the salinity stress tolerance in plants. Such studies have been carried out in halophytes such as *Thellungiella halophila* [19], *Suaeda aegyptiaca* [20], *Bruguiera gymnorhiza* [21], as well as in *Sesuvium portulacastrum* [22].

*Salicornia brachiata* Roxb and *Suaeda maritima* (L.) Dumort, belong to the Chenopodiaceae family [23,24]. They are herbaceous, annual halophytes widespread in the pichavaram mangroves, Tamil nadu, India. Both species have a succulent shoot and are known to be salt-accumulators growing well at a salt concentration of 200 mM NaCl [25,26,27]. Previous metabolomic studies, carried on *S. brachiata* and *S. maritima* treated with NaCl 200 mM and 500 mM, highlighted that these two halophyte species adopted different metabolic mechanisms to achieve acclimation to salinity. Anyway, in both species was observed an accumulation of osmoprotecctants (e.g., glycine betaine and polyols) suggesting that osmotic stress and oxidative imbalance adjustments were the key processes involved in NaCl tolerance [28]. Although Na^+^ accumulation, effects on plant growth, as well as metabolic changes induced by NaCl have been largely characterized [25,28,29,30], few studies have been focused on salinity-induced protein variations in *S.maritima* and *S. brachiata.* Furthermore, previous research indicate that these two species are expected to exploit different mechanisms to adapt to salinity.

Therefore, the aim of this study was to comparatively investigate, through a proteomic approach, the impact of NaCl on protein profile and content of *S. brachiata* and *S. maritima.* This information could provide an additional insight on plant salt stress tolerance, eventually supporting the scientific efforts to develop salt tolerant crop species.

## 2. Results

To investigate the effect of salinity, the changes at proteome level in *S. brachiata* and *S. maritima* treated with NaCl 200 and 500 mM were analyzed through a shotgun MS approach.

**Figure 1:**
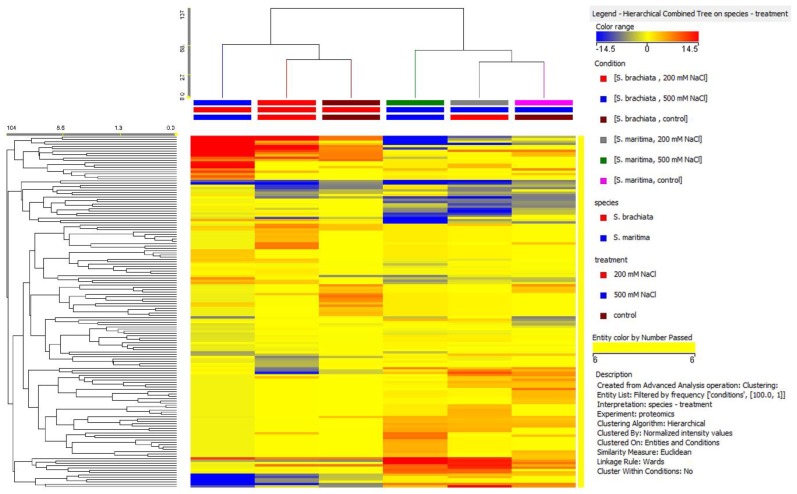
Unsupervised hierarchical cluster analysis of *Salicornia brachiata* and *Suaeda maritima* treated with 200 mM NaCl and 500 mM NaCl. Clustering was carried out on both conditions (treatments, vertical dendrogram) and compounds (proteins, horizontal dendrogram).

In a preliminary step, the unsupervised hierarchical cluster analysis were performed to have an overview of the protein profiling. The results pointed out that *S. brachiata* and *S. maritima* presented different profile based on proteins, as expected, since two main cluster separated both species (Figure 1). Moreover, 200 mM NaCl and 500 mM NaCl had different effect on plant proteins cauterizing in two different cluster within the species.

**Figure 2:**
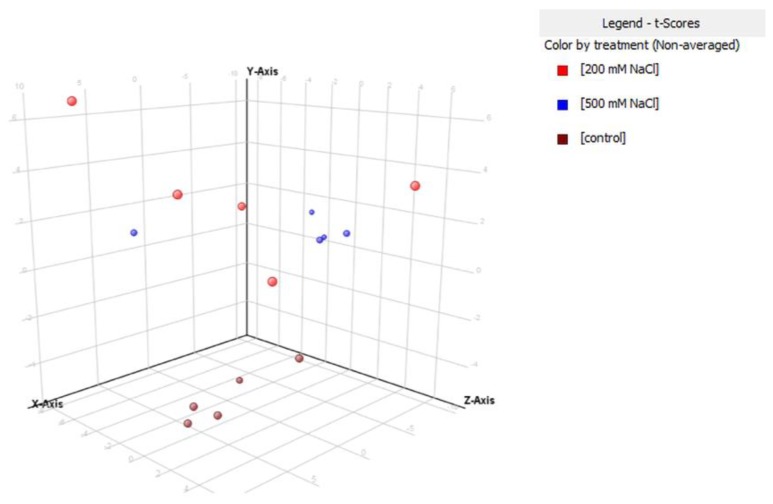
Partial least squares discriminant analysis (PLS-DA) on *Salicornia brachiata* according to their protein response to 200 mM NaCl and 500 mM NaCl. Individual replications are given in the class prediction model score plot.

**Figure 3:**
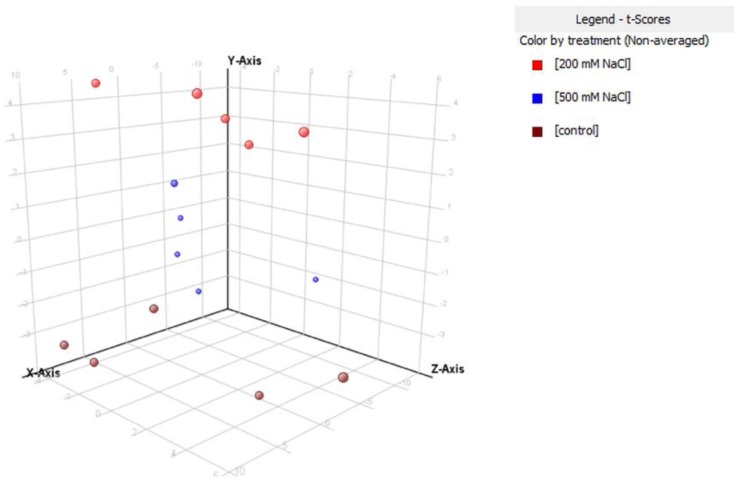
Partial least squares discriminant analysis (PLS-DA) on *Suaeda maritima* according to their protein response to 200 mM NaCl and 500 mM NaCl. Individual replications are given in the class prediction model score plot.

Overall, 150 proteins have been identified after validation of the results (false discovery rate = 1%). Significant features have been then identified through multivariate statistics, using the software Mass Profiler Professional (Agilent Technologies, Santa Clara, CA, USA). The whole datasets of proteins identified in *S. brachiate* and *S. maritima* are provide as supplementary material in Table S1 and Table S2, respectively. A multivariate partial least square discriminant analysis (PLS-DA) was subsequently carried out in *S. brachiate* (Figure 2) and *S. maritima* (Figure 3). This supervised analysis showed a clear separation between treatments being the accuracy of prediction 100%. Those proteins characterized by the highest score in the PLS-DA model underwent a fold-change analysis using a cut-off value of 5.

Functional annotation revealed that the proteins regulated by salt stress could be grouped into few categories. Regarding *S. brachiata*, photosynthesis, response to osmotic/oxidative stress, transcription, protein metabolism, cell wall and cytoskeleton, as well as signaling resultingly affected by salinity (Table 1). However, photosynthesis, energy and amino acids metabolism, cell structure, cell cycle, transcription, and protein metabolism were modulated in *S. maritima* (Table 2).

### 2.1. Photosynthesis and Energy Metabolism

In *S. brachiata*, cytochrome b6 that up-accumulated at both NaCl concentrations, the most of photosynthesis-related proteins (ATP synthase subunit alpha, apocytochrome f, cytochrome b6-f complex subunit 4) pointed out an up-accumulation at the lowest concentrations and a down-accumulation at the highest (Table 1).

In *S. maritima*, the level of cytochrome b6, apocytochrome f, photosystem I iron-sulfur center were up and down-accumulation at 200 and 500 mM NaCl, respectively. On the contrary, ribulose bisphosphate carboxylase large chains and ATP synthase subunits were down-accumulated at both salt conditions. The protein chloroplastic glyceraldehyde-3-phosphate dehydrogenase A, involved in energy metabolism, was down-accumulated at both NaCl concentrations, while cytosolic glyceraldehyde-3-phosphate dehydrogenase 2 showed a contrasting behavior being down-accumulated at the lowest NaCl concentration and up-accumulated at the highest. Finally, the level of pyruvate decarboxylase 1 was up-accumulated at both NaCl concentrations (Table 2).

### 2.2. Amino Acids Metabolism

Acetolactate synthase 1 was the protein, involved the amino acid metabolism, which was significantly affected in *S. maritima* pointing out an up- and down-accumulation at 200 and 500 mM NaCl treatments, respectively (Table 2).

### 2.3. Stress and Defense-Related Proteins

In *S. brachiata*, the proteins related to oxidative/osmotic stress such as heat shock protein 82, catalase isozyme 1 and 1-Cys peroxiredoxin PER1 were down-accumulated. On the contrary, the level of peroxidase 42 was up-accumulated under both salt conditions, whereas chaperonin CPN60-1 was up-accumulated at 200 mM NaCl but down-accumulated at 500 mM NaCl (Table 1).

### 2.4. Protein Folding and Degradation-Related Proteins

In *S. brachiata*, the proteins related to transcriptional regulation, homeobox protein HOX1A and ADP-ribosylation factor were up-accumulated at both salt treatments. Nonetheless, the proteins related to protein synthesis, turnover were also altered by salinity. The level of eukaryotic initiation factor 4A, luminal-binding protein 3 were up-accumulated at 200 mM NaCl but down-accumulated at 500 mM salinity, while the expression of histone H2A was down- and up-accumulated at 200 mM and 500 mM NaCl, respectively (Table 1).

In *S. maritima*, the proteins related to transcription like DNA-directed RNA polymerase subunit beta were up-accumulated at both concentrations. The level of histone H4 were up- (200 mM) and down-accumulated (500 mM) while protein HIRA was down (200 mM) and up-accumulated (500 mM) by NaCl treatments. Moreover, ribosomal proteins altered their abundance during salinity stress. In particular, at both NaCl concentrations the 60S acidic ribosomal protein P0 was up-accumulated, whereas the Ubiquitin-40S ribosomal protein S27a was down-accumulated (Table 2).

### 2.5. Cell Organization-Related Proteins

In *S. brachiata* the protein expansin-B1 was down-accumulated at both salt treatments, while the leucine-rich repeat receptor-like kinase protein, a protein promoting vegetative meristem, was down-accumulated. However, the protein terminal ear1 (regulating leaf initiation rate and shoot development) [31] was up-accumulated at 200 mM NaCl pointing out an opposite trend at the highest concentrations (Table 1).

In *S. maritima*, the level of Actin-1 was up and down-accumulated at 200 and 500 mM NaCl, respectively. Furthermore, an up-regulation of the gene encoding ‘Retinoblastoma’ (required for cell-cycle progression, endoreplication, transcriptional regulation, chromatin remodelling, and cell growth) was observed (Table 2).

### 2.6. Signaling-Related Proteins

Significant differences in signaling-related proteins were only observed in *S. brachiata*. Calmodulin, a Ca^2+^-dependent signalling molecule involved in the signaling network that mediates Na^+^ homeostasis and salt tolerance [32], was down and up-accumulated at 200 mM and 500 mM NaCl, respectively. On the contrary, indole-3-acetaldehyde oxidase, a protein involved in auxin biosynthesis, was up-accumulated at lowest concentration assayed and down-accumulated at the highest (Table 1).

## 3. Discussion

The adaptive response of plants to various abiotic stress is mainly interceded by profound alteration in gene expression, which leads to changes in composition of plant transcriptome, proteome and metabolome. A number of studies have previously reported that the changes in gene expression at RNA level does not always correlate with the protein level [33]. The analysis of alteration in plant proteome is significant because proteins are key regulators of cellular responses and are also mainly involved in stress tolerance. Therefore, a proteomic investigation provides a powerful tool to reveal the potential associations between the protein expression and plant stress acclimation. Thus, proteomics is helpful to achieve a better understanding of the plant system under saline environment. On this basis, we conducted a study focusing on the changes in protein profile of *S. brachiata* and *S. maritima* as triggered by NaCl (0, 200, 500 mM).

Among the metabolic processes altered by salinity, photosynthesis was one of the most affected. Salinity alters plant water uptake and induces a quick response in stomatal conductance through biosynthesis of abscisic acid and SOS signaling in leaves. This affects photosynthetic electron transport chain and the activities of enzymes involved in carbon fixation [34]. At the lowest concentration assayed (200 mM), the two species responded in a completely different manner. In particular, a generalized protein up accumulation was observed in *S. brachiata*. On the contrary, in *S. maritima* several proteins involved in photosynthesis (including RuBisCO and the chloroplastic glyceraldehyde-3-phosphate dehydrogenase A) were down accumulated by the treatment. Previous studies reported that 200 mM induced an increment of both fresh and dry weight in *S. brachiata*, suggesting that this salt concentration is optimal to improve its growth and development [25]. On the contrary, in *S. maritima*, concentrations ≥ 200 mM improved plant growth but the increase in fresh weight was mainly due to water accumulation rather than dry biomass production, indicating that this species activates physiological and biochemical mechanisms to tolerate salinity by mitigating and/or avoiding NaCl negative effects [35,36].

Among the proteins belonging to the photosynthesis and energy metabolism, cytochrome b6, Apocytochrome f and the chloroplastic ATP synthase subunit α have been identified in both species (*S. brachiata* and *S. maritima*). Previous studies demonstrated that cytochrome b6, and the chloroplastic ATP synthase subunit α play a pivotal role in response to several stresses [37]. During salinity stress, transpiration is reduced by stomatal closure and by a salinity-induced thickening of the mesophyll which reduces CO_2_ diffusion, as a consequence the availability of CO_2_ to the Calvin–Benson cycle might be limited [38]. Moreover, the increase of photorespiration cannot fully compensate for reduced CO_2_ availability as alternative sink for NADPH and ATP. In addition, either photorespiration or Calvin–Benson cycle might be repressed in response to both severe salinity and drought stress by a biochemical limitation of primary metabolism, even though RuBisCO levels are weakly affected by salinity [39,40,41]. Consequently, in response to long-term salinity stress, the electron transport of the photosynthetic machinery needs to be repressed, to avoid a large disproportion between the metabolic demand and the electron transport capacity. For example, during drought stress, *Helianthus annuus* strongly decreases the ATP synthase content and activity, reducing the ATP availability for RuBisCO regeneration and slowing down, as a consequence, the activity of the Calvin–Benson cycle [41]. Several studies focused on changes in electron transport and leaf assimilation capacity revealed a close correlation between cytochrome b6f complex and the electron transport capacity. In fact, it has been demonstrated that the inhibition of the cytochrome b6f complex induced a proportional decrease of both linear electron flux and leaf assimilation [42,43]. Moreover, during stress conditions and senescence, ATP synthase content was repressed in parallel in response to a reduced metabolic demand for ATP and NADPH [44,45].

On the other hand, only when the reduction in ATP synthase content is higher than 50% a linear reduction of the ATP synthase activity and assimilation could be observed [46].

In *S. brachiata*, cytochrome b6f complex and the chloroplastic ATP synthase subunit α were up accumulated at 200 mM NaCl whereas ATP synthase was weakly down accumulated at 500 mM NaCl. On the contrary, in *S. maritima*, both proteins where strongly down accumulated at the highest salt concentration (with chloroplastic ATP synthase subunit α having the same trend at 200 mM NaCl), suggesting a reduction of the photosynthetic activity. In fact, the decrease of ATP synthase levels indicates the disturbance in energy metabolism caused by salinity, as previously observed in other species such as *S. salsa* and *Thellungiella* [47]. Nonetheless, these findings are in agreement with the lower tolerance of *S. maritima* to NaCl, as compared to *S. brachiata*.

In addition to chloroplastic responses, the enzymes involved in glycolysis were also affected by salt treatments, but only in *S. maritima.* This effect was much less evident in *S. brachiata*, where a fold change lower than five was observed. Glycolysis is a pivotal pathway for energy production in plant cells. The alteration in the glycolytic enzymes NAD(P)H-dependent glyceraldehyde-3-phosphate dehydrogenase (GAPDH) and pyruvate decarboxylase (which, together with alcohol dehydrogenase, catalyse the decarboxylation of pyruvate to acetaldehyde and the reduction of acetaldehyde to ethanol) confirms the increase in catabolic energy production during salinity [48]. GAPDH is necessary to balance of cellular ATP levels and carbohydrate metabolism, thus affecting the energy status in plant and supporting its growth [49]. A down-regulation of the ATP synthase we observed at both concentration in *S. maritima*, and the down-regulation of both cytosolic and chloroplastic GAPDH further supports the lower tolerance to salinity of this species.

Plant stress adaptation is considered by an effectual adjustment of cellular metabolism to changing environmental conditions. During acclimation, the accumulation of numerous protective proteins and detoxification associated ROS scavenging enzymes occur to minimize the harmful effects of increased amounts of toxic by-products of altered cellular metabolism [17,50]. It is well known that one of the main targets of NaCl is the impairment of the mitochondrial function. Our results showed that in *S. brachiata* heat shock proteins (HSPs), which protect Complex I electron transport and might have a role in adaptation to NaCl [51], are affected by NaCl. In addition, Hamilton and Heckathorn [52] demonstrated that Complex electron transports could also be protected by antioxidants and metabolites as proline and betaine. In our previous experiments we demonstrated that 200 and 500 mM NaCl treatment strongly induced the production of several antioxidant compounds (flavonoids, phenolics etc) in *S. brachiata* as well as proline (only in roots), glycine betaine and polyols concentration, suggesting that the plant was able to cope with stress protecting cell from reactive oxygen species production [28]. In addition, our hypothesis is strongly supported by the significant up accumulation of the peroxidase, which is crucial for the detoxification of any excess H_2_O_2_ produced by SOD during salinity stress and which is known to increase under salinity [52].

Furthermore, salinity stress extremely affects the stability/composition of the cytoskeleton [53]. It has been proven that there is a strict positive correlation between the response of salinity-induced reduction of expansins, components of the cell wall, and plant growth [54]. In fact, they play an important role in increasing wall flexibility and in promoting leaf growth under drought and salinity stress [55]. In our experiments, expansin-B1 abundance was reduced, at both NaCl concentrations, only in *S. maritima,* whereas in *S. brachiata* it was not significantly affected.

As expected, cell signaling was affected by salinity treatments, through the modulation of secondary messengers (via both leucine-rich repeats receptor-like protein kinases and ADP-ribosylation factor ARF1). Plant leucine-rich repeats receptor-like protein kinases (LRR-RLKs), which play important roles in the signal perception, amplification and transduction to abiotic stress, were found to be down-regulated in *S. brachiata*. Changes in abundance of several cytoskeletal and cytoskeleton-associated proteins like actin and retinoblastoma-related protein, both necessary during cell cycle and cytokinesis, have been observed in salt treated plant cells [47,56,57,58,59]. In our experiment, ADP-ribosylation factor ARF1 was accumulated under both treatments in *S. brachiata*. It has been largely reported that ARF1 might play an important role in plant response to abiotic stresses. Joshi et al. [59] reported that transformed Arabidopsis and rice plants overexpressing the ARF1 gene, cloned from the halophyte *Spartina alterniflora,* where characterized by a strongly increased resistance to salinity and drought stress Similarly, also Karan and Subudhi [60] demonstrated the direct involvement of an ARF gene (namely SaARF) in abiotic stress adaptation in *S. alterniflora*. In particular, they observed that the SaARF gene was transcriptionally regulated in both leaves and roots under enhanced salinity and drought, and mediated tolerance to multiple abiotic stresses. Both experiments demonstrated that that transformed Arabidopsis and rice seedlings overexpressing SaARF where characterized by an increased tolerance to drought by maintaining chlorophyll synthesis, a high relative water content, membrane stability, and an increased biosynthesis of osmoprotectants.

In addition, [61] observed that in transformed tabacum plants the gene ARF1 conferred heat stress resistance and stimulated seed germination during adverse conditions.

The modulation of both transcription and translation was also outlined in our experiments at various levels. In fact, this latter level of regulation included alterations in histones, translational initiation factors and a homeobox protein levels. In eukaryotes, histone proteins are subjected to various post-translational modifications including acetylation, methylation, phosphorylation, ubiquitination, sumoylation, and ADP ribosylation [62]. In our experiments, histone H2A was down accumulated at the lower salinity dosage and up accumulated at the higher dosage in *S. brachiata*. However, histone H4 was up and down accumulated at the lower and the higher NaCl dosage respectively, in *S. maritima*. Previous studies have revealed that the occupancy of each histone variant of a core histone, in particular H2A and H3, plays important roles not only gene expression, but also in the repair of DNA breaks and the assembly of chromosome centromeres in eukaryotes [63,64,65]. Moreover, Sokol et al. [66] reported that both tobacco and *Arabidopsis* cells exposed to salinity stress showed the typical nucleosomal response that included histones modification. Eukaryotic translational initiation factor 4A (eIF4A) belongs to the family of helicases, important proteins involved in several cellular and metabolic processes including abiotic stress tolerance in plants [67]. Our results showed that eIF4A was up accumulated in *S. brachiata* at the lower dosage and down accumulated at higher dosage. The involvement of eIF4A genes in abiotic stress tolerance has been reported for several plant species like tobacco [68], rice [69], pea [70,71], and groundnut [72]. A transgenic approach highlighted the role of eIF4A genes in abiotic stress tolerance highlighted a reduced accumulation of sodium as primary mechanism [68,73]. Homeobox transcription factors are involved in various aspects of plant development, including the biosynthesis and signaling pathways of different hormones [74]. Our studies found that Homeobox protein HOX1A was up accumulated under salinity in *S. brachiata*. In literature, is reported that transgenic lines overexpressing HOX1A were characterized by an enhancement of gibberellin (GA) response [74]. These results are in agreement with our previous findings were an up accumulation of several gibberellins has been observed in *S. brachiata* treated with NaCl [28].

Furthermore, in our experiment, Luminal-binding protein 3 was up accumulated at the lower NaCl dosage and down accumulated at the higher NaCl dosage, in *S. brachiata*. This protein belongs to the HSP 70 superfamily and is involved in endoplasmic reticulum (ER) quality control mechanisms by recognizing unfolded or abnormally folded proteins thus avoiding their accumulation in the ER lumen [75] and the subsequent impairment of its secretory activity [76].

## 4. Materials and Methods

### 4.1. Plant Material and Salt Treatment

Seeds of *S. brachiata* and *S. maritima* were collected from Pichavaram, Tamil Nadu, India. The seeds of *S. maritima* and *S. brachiata* were germinated in vermiculite filled pots. The rooted plants were shifted to hydroponics containing modified Hoagland’s medium [36] and maintained at 25 ± 2 °C with a 16/8 h light/dark photoperiod. The plants were acclimatized for seven days in modified Hoagland’s medium and treated with 0 (control treatment), 200 and 500 mM NaCl for 14 days. The nutrient solution was replaced with freshly prepared solution at seven-day intervals.

The choice of the concentrations was based on previous experiments which demonstrated that the concentration 200 mM stimulated biomass production in both species whereas concentrations higher than 400 mM induced a reduction in plant growth and development [25,77]. On these bases, we decided to use NaCl at 0 mM as control treatment, 200 mM as optimal concentration and 500 mM as a moderate stress concentration. Harvesting (10 plants per treatment) was performed during daytime and the tissues were snap-frozen and powdered in liquid nitrogen to quench the endogenous metabolism and then stored at −80 °C for subsequent analyses.

### 4.2. Protein Extraction

Proteins were extracted as previously described by Lucini and Bernardo [48]. In Brief, 100 mg of powdered shoot tissues were suspended in 0.8 mL protein extraction buffer. An equal volume of Tris-buffered phenol (pH 8.0) was also added, then shaken well and centrifuged at 15,000× *g* for 5 min at 4 °C. The upper phase was transferred to the new tube and 5 volumes of precipitation solution (ice cold 0.1 M ammonium acetate in methanol) were added. The mixed solution was incubated overnight at −20 °C and centrifuged at 10,000× *g* for 15 min at 4 °C. The obtained pellet was then washed three times with 80% acetone (*v*/*v*) and air dried. Finally, the dried pellet was resuspended in a buffer containing 3 M urea and 2 M thiourea. Protein concentration was estimated, using the Bio-Rad protein assay kit according to the manufacturer’s instructions, and using bovine γ-globulin as standard. Protein samples were stored at −20 °C for further use. Finally, 50 μg of proteins were taken and reduced with dithiothreitol (DTT), alkylated with iodoacetamide and followed by an overnight digestion with trypsin (Promega, Madison, WI, USA) at 37 °C.

### 4.3. Proteomic Analysis by Tandem-MS

Tryptic peptides were analysed by means of a shotgun LC-MS/MS through a hybrid quadrupole-time-of-flight (Q-TOF) mass spectrometer. In particular, an Agilent 6550 IFunnel Q-TOF mass spectrometer, equipped with a nano-LC Chip Cube source (Agilent Technologies, Santa Clara, CA, USA) was used. The chip was composed by both a 40 nL enrichment column (Zorbax300SB-C18, 5 μm pore size) and a150 mm separation column (Zorbax300SB-C18, 5 μm pore size) coupled to an Agilent Technologies 1200 series nano/capillary LC system. All the instrument modules were controlled by the Mass Hunter Workstation Acquisition (Agilent Technologies, Santa Clara, USA) (version B.04).

Peptides were then loaded into the trapping column with a flow rate of 2.6 μL min^−1^ in using a solution composed by 2% acetonitrile (*v*/*v*) and acidified with 0.1% (*v*/*v*) formic acid. After the enrichment step, the chip was switched to separation mode and peptides were back flush eluted into the analytical column, during a 150 min acetonitrile gradient (from 3 to 70% *v*/*v*) in 0.1% (*v*/*v*) formic acid at a flow rate of 0.3 μL min^−1^. The mass spectrometer worked in positive ion mode and MS scans were acquired over a range from 300 to 1700 *m*/*z* at 4 spectra s^−1^. Precursor ions were selected for auto-MS/MS at an absolute threshold of 1000 and a relative threshold of 0.01% and considering a maximum of 20 precursors per cycle and an active exclusion set at two spectra (with release after 0.2 min). Afterwards, Spectrum Mill MS Proteomics Work bench (Agilent Technologies, Santa Clara, CA, USA) (RevB.04;) was used for the analysis of MS/MS spectra for peptides identification. Auto MS/MS spectra were extracted from raw data accepting a minimum sequence length of three amino acids and merging scans with the same precursor within a mass window of ±0.4 *m*/*z* in a time frame of ±30 s. Search parameters were: (a) Scored Peak Intensity (SPI) ≥ 50%, (b) precursor mass tolerance of ±10 ppm, and (c) productions mass tolerance of ±20 ppm. Carbamidomethylation of cysteine was set as fixed modification while trypsin was selected as digestion enzyme, accepting two missed cleavages per peptide. The search was conducted using the section “plants” in UniProt [78]; the database was concatenated with the reverse one. Auto thresholds were used for peptide identification in Spectrum Mill to achieve a target 1% false discovery rate. A label-free quantitation, using the protein summed peptide abundance was carried out following identification.

### 4.4. Statistical Analysis

The experiments were carried out in a completely randomized design with triplicates for reproducibility. The raw data on proteins signal intensities were imported into the Mass Profiler Professional B.04 (Agilent Technologies, Santa Clara, CA, USA) for multivariate statistical analysis. Protein intensities were log2 normalized and baselined versus the control. Afterwards, the dataset was elaborated through partial least squares discriminant analysis (PLS-DA) supervised multivariate statistics (N-fold validation = 5). The proteins having the highest discrimination score in first and second latent vector were finally exported from loading plots and then subjected to fold-change analysis (fold-change cut-off = 5).

## 5. Conclusions

Plant proteome analysis is postulated as a powerful tool to shed light onto the physiological response to environmental stresses, since proteins are key regulators of cellular responses. In our study, two distinct protein profiles were observed between the halophytes considered under NaCl salinity. These findings suggest diverse mechanism of resistance to NaCl in *S. brachiata* and *S. maritima*. Overall, 500 mM NaCl implied a stress situation for both plant species. Proteomics revealed that a key process for plant development such as photosynthesis was affected by salinity in both cases. However, *S. brachiata* showed a positive response to 200 mM NaCl, while *S. maritima* presented a down-accumulation of photosynthesis-related proteins after NaCl addition.

Although a coordinate proteomic response to salinity could be observed in both halophytes, the modulation of other stress-related processes was again different between *S. brachiata* and *S. maritima*. This coordinate response involved secondary processes crucial for plant survival, including plant signaling, heat shock proteins related to folding and mitochondrial electron transport chain, peroxidase, modulation of cytoskeleton (expansins), and regulation of both transcription and translation.

In addition to the distinct mechanisms adopted, the specific differences observed between the two halophytes corroborate the hypothesis that *S. brachiata* exhibits a higher tolerance to salinity, compared to *S. maritima*. These points deserve future attention, when halophytes are to be adopted as model plants to gain insight into plants tolerance to salinity.

## Figures and Tables

**Table 1:** Discriminant proteins in *Salicornia brachiata* shoots under either 200 mM and 500 mM NaCl salinity, together with fold-change (FC) values and regulation.

		Swiss-Prot ID	(200 mM NaCl) vs. (Control)		(500 mM NaCl) vs. (Control)	
Processes	Proteins		Log FC	Regulation	Log FC	Regulation
Photosynthesis	ATP synthase subunit α, chloroplastic	P0C2Z4	0.39	up	−1.30	down
	Cytochrome b6	P05642	4.37	up	20.05	up
	Apocytochrome f	P46617	1.28	up	−0.07	down
	Cytochrome b6-f complex subunit 4	P0C317	3.96	up	−0.64	down
Signaling	Calmodulin	P41040	−4.02	down	7.62	up
	Indole-3-acetaldehyde oxidase	O23887	3.69	up	−0.64	down
Osmotic/oxidative stress	Heat shock protein 82	Q08277	−4.47	down	−12.92	down
	Chaperonin CPN60-1, mitochondrial	P29185	1.77	up	−1.30	down
	Catalase isozyme 1	P18122	−10.88	down	−8.20	down
	Peroxidase 42	A5H453	0.20	up	14.16	up
	1-Cys peroxiredoxin PER1	A2SZW8	−6.81	down	−18.17	down
Cell structure	Leucine-rich repeat receptor-like kinase protein THICK TASSEL DWARF1	P0DL10	−7.01	down	−15.54	down
	Protein terminal ear1	O65001	4.54	up	−0.64	down
	Expansin-B1	P58738	−6.22	down	−7.07	down
Protein synthesis/turnover	Histone H2A	P40280	−3.64	down	11.17	up
	Eukaryotic initiation factor 4A	Q41741	0.01	up	−7.20	down
	Homeobox protein HOX1A	P46605	0.20	up	13.70	up
	Luminal-binding protein 3	O24581	0.24	up	−5.29	down
	ADP-ribosylation factor	P49076	4.14	up	12.74	up
Other	Uncharacterized protein ycf73	P0C310	7.79	up	−0.64	down

**Table 2:** Discriminant proteins in *Suaeda maritima* shoots under either 200 mM and 500 mM NaCl salinity, together with fold-change (FC) values and regulation.

		Swiss-Prot ID	(200 mM NaCl) vs. (Control)		(500 mM NaCl) vs. (Control)	
Processes	Proteins		Log FC	Regulation	Log FC	Regulation
Photosynthesis and Energy metabolism	Ribulose bisphosphate carboxylase large chain	P00874	−0.35	down	−1.06	down
	Glyceraldehyde-3-phosphate dehydrogenase A, chloroplastic	P09315	−0.60	down	−0.69	down
	Glyceraldehyde-3-phosphate dehydrogenase 2, cytosolic	Q09054	−0.01	down	0.68	up
	Cytochrome b6	P05642	3.33	up	−11.89	down
	Apocytochrome f	P46617	4.05	up	−11.04	down
	Photosystem I iron-sulfur center	P0C359	3.14	up	−2.90	down
	ATP synthase subunit α, chloroplastic	P0C2Y5	−0.15	down	−12.21	down
	Pyruvate decarboxylase 1	P28516	0.31	up	4.09	up
Amino acids metabolism	Acetolactate synthase 1, chloroplastic	Q41768	8.01	up	−2.72	down
Cell Structure	Actin-1	P02582	0.25	up	−14.96	down
Cell cycle	Retinoblastoma-related protein 1	Q9LKX9	0.27	up	0.38	up
Transcription	DNA-directed RNA polymerase subunit beta	P0C501	0.31	up	7.83	up
	Histone H4	P62787	0.29	up	−0.70	down
	Protein HIRA	Q32SG6	−0.35	down	3.54	up
Protein synthesis/turnover	60S acidic ribosomal protein P0	O24573	8.42	up	9.96	up
	Ubiquitin-40S ribosomal protein S27a	P27923	−0.78	down	−1.32	down
Other	CRS2-associated factor 1, chloroplastic	Q84N49	0.31	up	7.99	up

## Supplementary Materials

The following are available online at https://www.mdpi.com/2223-7747/9/2/227/s1, Table S1. Whole dataset of proteins identified in *Salicornia brachiata*. Table S2. Whole dataset of proteins identified in *Suaeda maritima.*
